# Delayed diagnosis of human immunodeficiency virus infection in a patient with non-specific neurological symptoms and pancytopenia: a case report

**DOI:** 10.1186/1752-1947-8-104

**Published:** 2014-03-24

**Authors:** Marcin Moniuszko, Andrzej Moniuszko, Justyna Puciłowska, Karolina Kisluk, Marta Jeznach, Anna Grzeszczuk, Robert Flisiak, Anna Bodzenta-Lukaszyk

**Affiliations:** 1 Department of Allergology and Internal Medicine, Medical University of Bialystok, Sklodowskiej-Curie 24A Street, Bialystok, 15-269, Poland; 2 Department of Regenerative Medicine and Immune Regulation, Medical University of Bialystok, Waszyngtona 13 Street, Bialystok, 15-269, Poland; 3 Department of Infectious Diseases and Hepatology, Medical University of Bialystok, Zurawia 14 Street, Bialystok, 15-540, Poland

**Keywords:** AIDS, Delayed diagnosis, HIV, Non-specific neurological symptoms, Pancytopenia

## Abstract

**Introduction:**

Both non-specific presentation and asymptomatic course of human immunodeficiency virus infection lead to undiagnosed long-term persistence of the virus in a patient's organism.

**Case presentation:**

Here, we present a case of a 31-year-old Caucasian man with non-specific neurological symptoms and pancytopenia, who was referred to an internal medicine ward for further diagnosis. Upon admission to our hospital, he denied any past risky behaviors and refused to have his blood collected for human immunodeficiency virus testing. Later, he eventually provided consent to conduct the human immunodeficiency virus test which turned out to have a positive result. The overall clinical pattern indicated an advanced-stage of acquired immunodeficiency syndrome, which contrasted with the history he had provided.

**Conclusions:**

This case report indicates the need to consider human immunodeficiency virus/acquired immunodeficiency syndrome diagnosis in patients with non-specific neurological and hematological disorders. Our report also demonstrates difficulties that can be experienced by the physician while trying to obtain both a clear history and consent to perform human immunodeficiency virus testing.

## Introduction

Delayed diagnosis of human immunodeficiency virus (HIV) infection increases death rates and the risk of onward transmission [1]. On the one hand, despite growing awareness, many physicians are still reluctant to consider a possibility of HIV infection in their patients. On the other hand, the stigma associated with HIV infection discourages many patients from seeking an appropriate diagnosis and treatment [2,3].

## Case presentation

A 31-year-old Caucasian man, suffering from dizziness, sensation of imbalance, weight loss of 10kg, night sweats, cough, fever, and general weakness which lasted for several weeks was admitted to the neurology unit of a local regional hospital. On admission, he presented a wide-based gait, a positive Romberg sign, hyperesthesia of the feet, symmetrically diminished plantar reflexes, and right eye blindness (due to injury 4 years prior to the current admission). Routine laboratory tests revealed leucopenia (white blood cell count of 2.01 10^3^/μL with 12% of lymphocytes) and macrocytic anemia (hemoglobin concentration of 10.8g/dL, red blood cells count of 3.37mln/mm^3^, hematocrit of 34.07%, mean corpuscular volume was 96.9fL). His platelet level was also below the normal range (72,000/mm^3^).

Computed tomography of his head demonstrated cortical-subcortical atrophy and malformation of his right eyeball. Neither intracranial bleeding nor malformation of his ventricular system was observed. Abdominal ultrasonography did not reveal any organ impairment.

He was referred to the clinical university hospital for further diagnosis of pancytopenia. In the Emergency Room, he was initially diagnosed with dizziness, imbalance, and gait disorder along with leucopenia and was admitted to the internal medicine ward.

He confirmed a 10kg weight loss during the last several weeks that was supported by a physical examination. His arterial pressure, heart rate, and body temperature were 130/80mmHg, 70 beats per minute and 38°C, respectively. In addition, he presented hyperesthesia of the abdominal wall and both feet.

A chest X-ray revealed the presence of several inflammatory-like opacities in his left pericardial area and right upper lobe. The complete blood cell count revealed macrocytic anemia, leukopenia (720 cells/mm^3^) and mild thrombocytopenia (104,000/mm^3^; Table 1). His liver function was impaired with an increased aspartate and alanine aminotransferase along with gamma-glutamyltransferase (GGT) activities (351IU/mL, 167IU/mL, 281IU/L, respectively). Further laboratory tests revealed mild hypomagnesemia, hypoalbuminemia, elevated levels of beta- and gamma-globulins, and D-dimers. His level of complement 3 was below normal range (745mg/L) and 50% hemolytic complement activity of the serum was critically low. None of the anti-nuclear antibodies were found to be positive. His vitamin B12 levels were normal.

**Table 1:** Laboratory test results

**Parameter**	**Patient’s laboratory test results**		**Reference ranges**
**Red blood cell count**	**3.94 × 10**^**6**^**/mm**^**3**^		**4.5-6.0 × 10**^**6**^**/mm**^**3**^
**Hemoglobin**	**12.4g/dL**		**14–18g/dL**
**Hematocrit**	**38.1%**		**40–54%**
Mean corpuscular hemoglobin	31.6pg/cell		27–34pg/cell
**Mean corpuscular volume**	**96.9fL**		**80–94fL**
**White blood cell count**	**3.68 × 10**^**3**^**/mm**^**3**^		**4–11 × 10**^**3**^**/mm**^**3**^
Neutrophils relative count	70%		40–72%
Lymphocytes relative count	19.5%		18–48%
Monocytes relative count	7.6%		4–8%
Eosinophils relative count	1.4%		0.5–6%
Basophils relative count	0.4%		0–1%
**Platelet count**	**102 × 10**^**3**^		**130–350 × 10**^**3**^**/mm**^**3**^
Sodium concentration	136mmol/L		136–145mmol/L
Potassium concentration	3.6mmol/L		3.5–5.1mmol/L
**Calcium concentration**	**2.19mmol/L**		**2.25–2.75mmol/L**
**Magnesium concentration**	**0.78mmol/L**		**0.8–1mmol/L**
**Alanine aminotransferase**	**167IU/L**		**5–50IU/L**
**Aspartate aminotransferase**	**351IU/L**		**5–50IU/L**
**Gamma-glutamyltransferase**	**281IU/L**		**10–75IU/L**
Bilirubin – total	0.67mg/dL		0.2–1.2mg/dL
Albumin	40.5%	3.08g/dL	54.3–65.5%
**Alpha 1**	**4.4%**	**0.33**g/dL	**1.2–3.3%**
Alpha 2	9.1%	0.69g/dL	8.3–15%
Beta 1	8.1%	0.62g/dL	6.5–11.5%
**Beta 2**	**12.3%**	**0.93**g/dL	**2.5–7.2%**
**Gamma**	**25.6%**	**1.95**g/dL	**7.1–19.5%**
Total protein	7.6g/dL		6–8g/dL
Prothrombin time	109%		70–120%
International normalized ratio	0.95		
Fibrinogen	359mg/dL		200–400mg/dL
Activated partial thromboplastin time	31.1 seconds		24–35 seconds
D-dimer	0.84ug/mL		0–0.5ug/mL
C-reactive protein	<0.5mg/L		<10mg/L
**Complement C3**	**745mg/L**		**970–1576mg/L**
Complement C4	183mg/L		162–445mg/L
Procalcitonin	0.1ng/mL		<1.0ng/mL
Immunoglobulin M	223mg/dL		63–277mg/dL
Immunoglobulin A	**1440mg/dL**		**82–453mg/dL**
Immunoglobulin G	**1780mg/dL**		**751–1560mg/dL**
Vitamin B12	667pg/mL		187–883pg/mL
Alkaline phosphatase	98IU/L		37–110IU/L
Prostate-specific antigen	0.1651ng/mL		0–4.0ng/mL
Thyroid-stimulating hormone	0.7575μIU/mL		0.35–4.94μIU/mL
Lactate dehydrogenase	353U/L		200–480U/L
Folic acid	6ng/mL		2.7–34ng/mL
Rheumatoid factor	Negative		
Waaler–Rose factor			
Anti-ribonucleoprotein antibody			
Anti-Smith antibody			
Anti-SSA antibody			
Anti-double-strand (native) deoxyribonucleic acid			
Anti-SSB antibody			
Ro52 antibody			
Anti-PM-Scl antibody			
Proliferating cell nuclear antigen			
Anti-Jo-1 antibody			
Anti-mitochondrial antibodies – M2			
Antigen of the hepatitis B virus	Negative		
Antibodies against hepatitis B surface	Negative		

Results out of the normal range are highlighted in bold.

The patient was consulted on by an ophthalmologist and neurologist. Following the consultation, a cerebrospinal fluid (CSF) analysis was performed. The examined sample was colorless and clear, with cytosis of one cell and total protein concentration of 21mg/100mL. The result of a Nonne–Apelt test was negative.

Tests for toxoplasmosis, syphilis, and Lyme disease returned negative results. Tests for cytomegalovirus infection revealed enhanced antigen-specific immunoglobulin G (IgG) antibodies. A throat swab culture revealed the presence of *Escherichia coli*. Cultures of his CSF and sputum revealed the presence of *Kocuria varians/rosea* and *Staphylococcus aureus*, respectively.

For the first 2 days of hospitalization, the patient denied any past risky behavior and he refused HIV testing. Nonetheless, following repetitive supportive advice, he eventually provided consent to conduct an HIV test. The results of two consecutive enzyme-linked immunosorbent assay tests were positive along with further Western Blot testing. He reluctantly admitted his only risky behavior was intravenous illicit drug use 3 months prior to hospitalization. His CD4 cell counts were found to be significantly decreased (24 cells/mL). The overall clinical pattern indicated advanced-stage acquired immunodeficiency syndrome (AIDS), the diagnosis that stayed in contrast to the information provided by the patient.

He was referred to the Department of Infectious Diseases and Hepatology for further treatment, where he admitted having used intravenous drugs for 5 years prior to the development of the symptoms.

On admission, his viral load was 703,475 copies/mL and his CD4+ T-cell count was 24 cells/mL. In addition to previous findings, he was anti-hepatitis C virus (HCV) positive and HCV ribonucleic acid was detectable (HCV genotype 3). Further, antibodies against *Mycoplasma pneumoniae* were also present in the serum. Highly active antiretroviral treatment (HAART), zidovudine/lamivudine and nevirapine, was introduced without delay and resulted in clinical improvement along with CD4 normalization. Currently, 3 years later, the patient remains under ambulatory care. A family epidemiological investigation revealed HIV (the same subtype) and HCV infection of his wife and gave negative results in their 5-year-old daughter.

## Discussion

This case illustrates the possibility of HIV infection in patients with non-specific neurological symptoms accompanied by abnormal complete blood cell counts.

On admission, the combination of reluctantly presented inconsistent history (including acknowledging alcohol use), his relatively good appearance, neurologic symptoms, macrocytic anemia, thrombocytopenia, elevated liver enzymes (including GGT), hypomagnesemia, and hypoalbuminemia could have been suggestive of chronic alcohol abuse. This could have been one of the reasons for which he was initially referred to a neurologic ward and an internal medicine ward. Therefore, it is also possible that due to the apparently convincing clinical picture, the strategy of previous history taking did not take into consideration the possibility of retroviral infection. However, following the detection of anti-HIV antibodies, the patient modified his history stating that his only risky behavior was associated with episodic illicit drug use 3 months earlier, which was in contrast to his neurological symptomatology, which is much more frequent in late AIDS rather than during acute retroviral syndrome. Moreover, there is a direct relationship among neurological complications, phase of HIV infection, and the level of immunological involvement [4,5]. Although it is rare, the neurologic symptoms could be present during the acute phase of HIV infection [6,7]. Nonetheless, cases of patients with AIDS presenting only with isolated neurologic symptoms remain uncommon [8,9].

One of the surprising findings in our patient was identification of *Kocuria sp*. in his CSF. Nevertheless, he did not present any of the meningeal signs, such as neck stiffness or Kernig sign. *Kocuria sp*. is generally non-pathogenic; however, it was considered a causative agent of numerous cases of intracranial abscesses, meningitis, pneumonia, and septic arthritis in both immunosuppressive and immunocompetent individuals [10]. Although it is more probable that *Kocuria sp.,* in the CSF of our patient, was merely a sign of immunodeficiency rather than agent accounting for neurologic symptoms, the latter cannot be excluded.

Congruent with the complex history of our patient, pancytopenia is not a common sign of HIV infection. The literature concerning prevalence of pancytopenia in HIV-infected patients remains scarce. By contrast, anemia remains a frequent abnormality in these individuals. However, as opposed to the case of our patient, HIV infection is accompanied by normocytic rather than macrocytic anemia. As an example, in one early series of patients with AIDS, anemia was noted in approximately 70%, lymphopenia in 70%, neutropenia in 50%, and thrombocytopenia in 40% of people living with HIV [11]. Even in the absence of other pathological processes, bone marrow morphology is consistently abnormal: hypercellularity, dysplasia, plasmacytosis, and lymphoid infiltrates are frequently observed [11]. According to studies performed in African antiretroviral therapy -naive patients, severe anemia was present in 80% of cases. However, red cell morphology was variable with the majority normochromic and normocytic (64%) and 36% showing hypochromia and anisopoikilocytosis [12].

Similarly, the occurrence of thrombocytopenia is not very common in the course of HIV infection. A low platelet count can be caused by multiple mechanisms including direct effects of HIV on bone marrow megakaryocytes, immune-mediated destruction, decreased platelet production, effects of drugs on platelet progenitors or by the development of a form of thrombotic thrombocytopenic purpura [13]. According to the data derived from Collaborations in HIV Outcomes Research/US Cohort and the GlaxoSmithKline HIV Clinical Trials, thrombocytopenia affects 10 to 14% of patients [14]. Collaborative follow-up analysis performed in these two trials revealed that the occurrence of severe thrombocytopenia was rare; however, in 23% of the patients, the platelet counts remained unchanged, despite the introduction of HAART.

One of the crucial problems illustrated by our case is a delayed diagnosis of HIV infection in a patient with AIDS-related symptoms. Unfortunately, the problem of late HIV diagnosis is not only restricted to developing countries. A recent multicenter study, performed in Germany, revealed a delayed HIV diagnosis in as many as 49.5% studied individuals [15]. Similarly, in a study of 3000 Danish individuals diagnosed with HIV infection between 1995 and 2009, 51.2% were late presenters [16]. Recently, in concordance with this pattern, as many as 55.2% out of 7300 newly diagnosed Italian HIV-infected individuals were classified as late presenters; among these, 37.9% were diagnosed with AIDS [17]. In a recent study of an HIV-infected cohort for England and Wales, a late diagnosis has been described as the most important predictor of dying of AIDS: 60% of all-cause mortality and 81% of all AIDS-related deaths were attributable to a late diagnosis [1].

Altogether, a delayed HIV diagnosis has become an increasing challenge to worldwide healthcare systems. One of the possible causes of this problem is an insufficient level of HIV/AIDS awareness. Similar to the patient described in our report, many HIV-infected patients remain unaware of their HIV status. It is estimated that one-third of infected individuals are unaware of their disease, and not only do they not benefit from early treatment but they can transmit HIV to their sexual partners [18]. Some (like the patient described here) refuse to undergo HIV tests, declaring fear of HIV infection [19], fear of disclosure [20], and low-risk perception combined with selective HIV transmission knowledge [21]. Another consequence of insufficient knowledge on HIV transmission is the existence of HIV/AIDS stigma in many societies [2]. Such a stigma was documented as a barrier to undergo HIV testing in numerous settings, particularly in resource-limited countries [2]. In a study of 112 patients receiving antiretroviral therapy in Botswana, 2 years before the implementation of universal access to treatment, 69% of the patients did not disclose their HIV status to their family, and a majority of those who reported delayed testing for HIV did so due to fear of HIV/AIDS stigma [3]. However, we demonstrated here that a patient’s fear of learning his or her HIV status could be successfully overcome by health care workers providing psychological support .

## Conclusions

This case report suggests the need to consider HIV/AIDS diagnosis in patients with non-specific neurological and hematological disorders, and highlights complications associated with delayed HIV testing. Our report also demonstrates difficulties that can be experienced by the physician while trying to obtain both a clear history and consent to perform HIV testing.

## Consent

Written informed consent was obtained from the patient for publication of this case report and any accompanying images. A copy of the written consent is available for review by the Editor-in-Chief of this journal.

## Competing interests

The authors declare that they have no competing interests.

## Authors’ contributions

MM diagnosed the patient, provided and analyzed clinical data and drafted the manuscript; AM analyzed clinical data and drafted the manuscript; JP and KK analyzed clinical data; AG and RF diagnosed and treated the patient; MJ and AB-L helped to diagnose the patient, provided and analyzed clinical data. All authors reviewed and approved the final version of the manuscript.
